# Protein Complexes Help Point Migrating Cells in the Right Direction

**DOI:** 10.1371/journal.pbio.0040058

**Published:** 2006-01-24

**Authors:** Liza Gross

Self-generated movement is a key aspect of cellular life. From a single-celled amoeba hunting for food or mates to a human neutrophil finding and killing an infectious microbe, cells use many of the same molecular components to move.

Directed motility results from asymmetric activities in the cell, as localized signaling networks produce different physical and morphological changes at a cell's leading and trailing edge. This type of polarity occurs when chemical signals—associated with food, pheromones, bacteria, or antigens, for example—trigger and amplify internal signaling cascades that recruit proteins at polar ends of the cell to organize different actin structures at each end.

To modify its actin cytoskeleton in response to these chemoattractants, a fast-moving cell like the neutrophil must make one set of actin assemblies in front and another, completely different set in the rear, using different signaling pathways at each site. At the front, actin-rich structures form protrusions that project the cell forward. At the rear, actin–myosin contractile complexes retract the cell's trailing edge, helping it keep pace with the leading front edge. These structures and signaling pathways must be segregated so the assemblies can move in a coordinated manner. Segregation is reinforced by signaling pathway feedback loops, which in turn are likely controlled by specifically targeted regulatory protein complexes. Many questions remain, however, about the identity and function of the proteins that generate and maintain this polarity during chemotaxis. In a new study, Orion Weiner, Henry Bourne, Marc Kirschner, and their colleagues describe a family of protein complexes that regulate polarity circuits at the leading edge of migrating neutrophils.

One important regulator of polarity and cytoskeletal remodeling during chemotaxis is a protein called Rac. Neutrophils use Rac to control the positive feedback loops that stabilize its leading edge and Rho to oversee the myosin-driven contractions that bring along the rear. Rac induces actin polymerization under the direction of WAVE proteins, which mediate actin rearrangements and cell migration in processes as diverse as development and tumor metastasis. Rac and WAVE indirectly interact through a multicomponent protein complex called the WAVE regulatory complex. [Fig pbio-0040058-g001]

**Figure pbio-0040058-g001:**
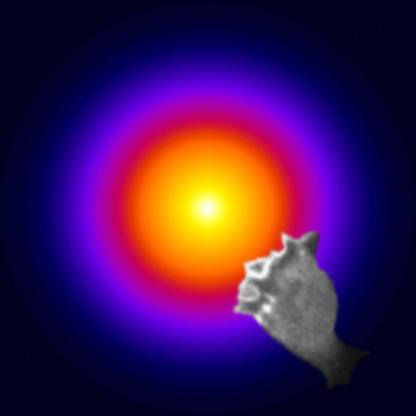
A neutrophil migrating toward a chemical gradient

Working with a neutrophil-like cell line, the authors focused on one of these components of the WAVE regulatory complex, Hem-1. The authors tagged Hem-1 with fluorescent protein to monitor its movements. In the absence of a chemical signal, Hem-1 is seen throughout the cytosol. But when a chemoattractant stimulates the cell, the proteins rapidly travel to the periphery, then accumulate at the leading edge as the cells polarize.

The authors suspected that Hem-1 complexes might regulate other proteins in addition to WAVE. If so, Hem-1 should exist in higher concentrations than WAVE2 (the WAVE protein most abundant in neutrophils), exist in distinct biochemical milieus, and associate with other proteins. Biochemical assays confirmed these predictions and showed that over 60% of Hem-1 did not associate with WAVE2, making a strong case that Hem-1 targets other proteins.

To find out what those targets might be, Weiner et al. isolated complexes containing Hem-1 to identify their components. As expected, they retrieved other members of the WAVE regulatory complex, but also found “excellent candidates” for regulating the positive and negative feedback loops required for neutrophil polarity, including proteins that could keep myosin complexes from acting in the front of the cell. The most abundant candidates likely associate with Hem-1 complexes that do not contain WAVE2, the authors concluded, because the majority of Hem-1-containing complexes do not associate with WAVE2.

Reducing Hem-1 concentrations in the neutrophils produced defects in actin polymerization, cell polarity, and morphology in the depleted cells, demonstrating Hem-1's essential role in these processes. Given Rac's importance in actin polymerization and cell polarity orchestration, the authors analyzed its activity in Hem-1-depleted cells. They found that Hem-1 complexes, which act downstream of Rac, are also required for Rac activation. This is consistent with a Rac positive feedback loop in which Rac increases its own activation via Hem-1 complexes. Positive feedback loops are highly conserved elements in cell polarity circuits from yeast to slime molds to neutrophils. Hem-1 supports this feedback loop through mechanisms that operate independently of and in addition to WAVE-regulated actin assembly. The authors go on to show that Hem-1 also protects the leading edge from the contractile influence of myosin.

Altogether, these results suggest that Hem-1 protein complexes function to organize the leading edge of migrating neutrophils. Here, these versatile complexes steer multiple inputs, such as Rac, to the appropriate output at the leading edge—whether it be stimulating WAVE-regulated actin polymerization, promoting feedback loops that stabilize the protrusions, or segregating activated myosin to ensure proper chemotaxis. The authors' next round of experiments will investigate the upstream signals that regulate the targets of Hem-1 complexes and help the cell reach its destination.

